# Notes and News

**Published:** 1890-03-15

**Authors:** 


### Notes and News.

Collected and Communicated.

Through an error, we lately stated that the Middlesex Hospital treated 3,000 patients last year, but the figures given in the report were 41,778, exceeding by 3,000 the largest number ever before recorded. The chapel also (which has been designed by Mr. J. L. Pearson, R.A.) is not yet begun, and was not, therefore, "completed in November last."

We hear that the following new members have joined the Hospitals Association: Mr. E. Murray Ind, Chairman of the London Hospital; Mrs. A. M. Francis, Matron of the Glamorgan and Monmouthshire Infirmary, Cardiff; Miss A. Griffiths, Matron of the Lambeth Infirmary; and Mr. Frederick Mitchell, Honorary Secretary of the Gravesend Hospital.

A letter reached our office this week from the Glamorgan and Monmouthshire Infirmary, and we were much struck by the excellent little picture of the infirmary at the top of the sheet of writing paper. The sketch, though small, showed at a glance that the infirmary was an important building, well constructed, with consideration both for beauty and utility. We advise all hon. secretaries to have their paper headed in this manner, if they wish to convey a truthful view of the importance of their institution to the minds of their correspondents.

The Paisley Infirmary had over 1,000 in-patients last year. Of these, 977 were cured, improved, or dismissed; 83 died, and 95 were still in the Infirmary on January 1st, 1890. Of the 83 patients who died, 14 were moribund when admitted. During the year, 5,350 patients were prescribed for at dispensary, an increase of 1,326 over the preceding year. It is gratifying to find that the income of the institution as a whole has been much larger than in the preceding year. The widow of the late Dr. Taylor has offered the Infirmary £500 towards building a ward to be named after her late husband. Mrs. Arthur has also given over £2,000 to the Infirmary. On the whole the report is very satisfactory; so is that of the Paisley Convalescent Home, which is just issued, and which tells of 693 persons benefited in the past year. Paisley is to be congratulated on its medical charities.

A donation to the National Pension Fund for Nurses comes from a lady lately operated on in one of our large hospitals. She says: "I am sorry I cannot send more, but being still an invalid I have to be careful of my little income; still, I felt I must give something, having received so much care from the nurses of Guy's. I am so thankful large contributions are being given to the fund, for the kindness and attention I received from everyone shows me how worthy hospital workers are."

The German Hospital in Philadelphia is notable because, in spite of its name, it is in no way exclusive. While known as the "German" Hospital, it makes no distinction as to nationality, creed, or colour, and the larger number of patients admitted are English-speaking people. Last year, to every 68 English-speaking persons treated there were 40 Germans and 21 persons of other nationalities. The total number of patients during the past year was 5,814, of whom 1,678 were admitted to the hospital, and 4,136 were attended to in the dispensaries. The accident cases received numbered 1,713, and of these 404 were admitted to the hospital wards. The nurses are all Sisters of the Mary J. Drexel Home, and they take great pride in looking after the wants of the institution and the care of the patients.

Microbes as a race are doomed, of a surety. A "fiercer light than ever beat upon a throne" is turned upon the interesting little demons—the eye of science, that is. One by one, are they dragged out from that darkness—of ignorance—in which their evil deeds have for so long flourished. The latest to make its *début* in public is that of the small-pox, which can, according to Dr. Sicard of Beziers, be readily cultivated on "gelatine that has been sterilized by bichloride of mercury." This particular bacterion belongs to the class of cocci; in appearance it is round, with "a transparent centre, umbilicated in the middle, while its edges are raised." It can be easily found at all stages of the small-pox, in the pustules of the skin, in the mucus of the bronchial tubes, and in the blood. More than that, it hangs suspended in the air of a room containing a small-pox patient. If this be so, there can be small difficulty in inoculating society in general with the real Simon Pure. As it is, however, those animals "already inoculated with cultures of this bacterion," although evincing certain symptoms, "show no relation to variola, either in its form or evolution!"

Grimsby is going in for a clean sweep and a fresh staff, and hopes by this means to reduce its expenditure to less than £100 per bed. For a number of years the Grimsby Hospital has been deep in debt, and for many years the expenditure has far overtopped the receipts, and notwithstanding that at the time of the jubilee a special fund to ease its burden was secured, yet things have gone from bad to worse. Economy has been practised, but still the hospital has sunk deeper into debt, and at the present time has a burden of £900. An investigation committee was recently formed to go into the matter of the income and outgo, and the services of three matrons of other institutions were obtained. The whole question was laid before them, and they attended personally to inspect the hospital and prepare a report. From the report it appears that enormous sums have been expended in surgical instruments and appliances, and that the staff is much too large. Not only so, but it was demonstrated that the hospital could be managed in such a way as to bring the expenditure within the income. The report is a remarkable one, and proves that mismanagement has been the cause of the indebtedness of the institution. The house surgeon and matron have both sent in their resignations, and the whole of the remaining staff will be discharged.

The following vacancies are announced this week, the amount of the salary in each case being given after the name of the institution:

**Resident Medical Officers.**

Alnwick Infirmary, House Surgeon—£120, rooms, etc.
Birmingham City Hospital, Clinical Assistant—Board, etc.
Wallasey Dispensary, Assistant House Surgeon—£80, rooms, etc.
West London Hospital, House Surgeon.
Jessop Hospital for Women, House Surgeon—£50, board, etc.
Royal Berks Hospital, Assistant House Surgeon—£40, board, etc.
Morpeth Dispensary, House Surgeon—£170, rooms, etc.

**Non-Resident Medical Officers.**

County Council of Lancaster, Medical Officer of Health—£800.

Durness, Sutherland, Medical Officer—£150.

Kensington Dispensary, Honorary Medical Officer.

Wolverhampton Union, Medical Officer of District—£140.

Parish of Fettercairy, Medical Officer—£18.

Truro Union, Medical Officer of Health—£45.

St. George's and St. James' Dispensary, Physician and Surgeon.

University of London, Examiner in Surgery—£150.

A query about the School of Science, Trinity College, Wallington, and St. John's College, Penge, which appeared in our pages last week, seems to point to more doubtful diplomas being offered to the public. Certainly several people who have lived long in Wallington have never heard of Trinity College there, and the School of Science does not appear in directories or dictionaries of London.

The new Hull Victoria Hospital for Children was commenced on February 22nd, when Mrs. Arthur Wilson laid the foundation stone. The Mayoress and Mrs. Wilson afterwards held a reception at the Artillery Barracks. So long ago as 1883, the old Children's Hospital was voted overcrowded, and it was decided to secure a new and a larger site for the erection of a thoroughly good hospital; but it was not till 1886 that a desirable piece of ground was found and bought. Then there was considerable difficulty in raising funds, though Mr. Arthur Wilson led the way with a donation of £1,000. Even now another £2,000 is required to open the building free from debt. Hull has had a hard struggle to obtain its Children's Hospital; we hope they will be able to maintain it efficiently when completed.

Mr. John Deacon presided on Tuesday at the annual meeting of the Royal London Ophthalmic Hospital, Moorfields. Very few legacies were received last year, and the committee were obliged to sell out £2,000 worth of Consols to meet the ordinary expenses. This was very hard, as they are in treaty for a neighbouring piece of ground on which they had hoped to build a new hospital at a cost of £50,000. Under the circumstances, the committee have decided to confine the enlargement of the building to spending £10,000 on the piece of ground at the back, and towards this sum donations are invited. Over 29,000 patients were treated last year.

There is peace at last at the Suffolk General Hospital, thanks to the efforts of the Duke of Grafton and Mr. Bunbury. The chief proposals which have brought about this happy state of affairs are the following: "That there be three honorary medical officers appointed to the charge of the hospital patients, and one honorary assistant officer. The three medical officers to have entire charge of the in-patients, and to attend to the out-patients subject to the exigencies of their private practice. They shall each have the choice of the days and hour most convenient to them for seeing out-patients, with the distinct understanding that Wednesday must be one. The three medical officers to be *ex-officio* members of the Weekly Committee with full powers. That the Revision Committee be strengthened by the addition of the three medical officers."

We are informed that Mr. Gladstone has kindly consented to open the new Residential Medical College at Guy's Hospital, on Wednesday, March 26th, at 2 p.m.

The Hospital Saturday Fund as a body enrolled under the Companies Acts held its first meeting lately at the offices, Mitre Court, Fleet Street. Delegates were nominated for the new finance, distribution, and workshop and street collection committees, the election for which will take place at the next meeting. The date of Hospital Saturday for the present year was fixed for July 12.

There was great excitement at Chester Infirmary over the election of Honorary Physician last week. The Duke of Westminster wrote: "Eaton is full of influenza just now, and though the children are recovering, we are advised it is necessary to keep away a bit longer, so that I am unable to attend the meeting on Tuesday, which I much regret. Sir Henry Roscoe and Mr. Jaffray, of Birmingham, have recommended Dr. Elliott very highly, but more than this I am unable to say, as I do not know the qualifications of the candidates." But in spite of this letter Dr. King was elected by a large majority. An interesting point was raised by Mr. Thompson with regard to the infirmary nurses and the nurses of the Deaconess Institution. It is proposed to send out private nurses from the Infirmary, which Mr. Thompson objected to. He said: "At the Deaconess House they had not a single nurse who was not certified perfectly efficient, besides which they had the testimony of the medical men of the city as to their efficiency and to the absolute necessity for the institution. He thought he was justified therefore in saying that anything tending to the removal of these nurses would be nothing short of a calamity. Miss Vickers, the lady superintendent, had also been sent to an institution in London that she might perfectly train the district nurses. With this question of private nursing, however, came the danger that if they were to be taken away it would defeat the object of the Deaconess Institute, as their funds would fail and the district nursing must naturally be given up. One of the great disadvantages, he was afraid, was that the nurses were not trained at the Infirmary; but that was no fault of the Governors of the Deaconess Institute. One fatal objection was that they did not give lectures at the Infirmary, and the nurses' diplomas were not complete in consequence." Of course the answer to all this is obvious; the Infirmary ought to give its nurses lectures and train them for the Deaconess Institute, and it ought not to interfere with the private nurses of the Institute, whose earnings help on the district nurses who attend to the poor.

